# RETRACTION: Genetic Clearness Novel Strategy of Group I *Bacillus* Species Isolated from Fermented Food and Beverages by Using Fibrinolytic Enzyme Gene Encoding a Serine‐Like Enzyme

**DOI:** 10.1155/jna/9801841

**Published:** 2026-03-25

**Authors:** Journal of Nucleic Acids

RETRACTION: Kaya‐Ongoto, M.D, Kayath, Christian A, Nguimbi, E, Lebonguy, Aimé A, Nzaou, S.A.E, Elenga W, Paola S, Ahombo, G, Genetic Clearness Novel Strategy of Group I *Bacillus* Species Isolated from Fermented Food and Beverages by Using Fibrinolytic Enzyme Gene Encoding a Serine‐Like Enzyme, *Journal of Nucleic Acids*, 2019, 5484896, 13 pages, 2019. https://doi.org/10.1155/2019/5484896.

The above article, published online on 20th May 2019 in Wiley Online Library (http://wileyonlinelibrary.com), has been retracted by agreement between; the journal Editor, Kalyani Sen; and John Wiley & Sons Ltd. The retraction has been agreed due to figure concerns. Specifically, there are multiple duplicated bands within Figure 4.

The results are therefore considered unreliable, and the article is retracted.

The authors disagree with the retraction.

**References**

[1] Actinopolyspora biskrensis, “Genetic Clearness Novel Strategy of Group I Bacillus Species Isolated from Fermented Food and Beverages by Using Fibrinolytic Enzyme Gene Encoding a Serine‐Like Enzyme” *PubPeer,* Nov 2022, https://pubpeer.com/publications/E67D58189E6083CE099FCF37535A6A

